# Using Generative Artificial Intelligence Tools in Cosmetic Surgery: A Study on Rhinoplasty, Facelifts, and Blepharoplasty Procedures

**DOI:** 10.3390/jcm12206524

**Published:** 2023-10-14

**Authors:** Bryan Lim, Ishith Seth, Skyler Kah, Foti Sofiadellis, Richard J. Ross, Warren M. Rozen, Roberto Cuomo

**Affiliations:** 1 Department of Plastic and Reconstructive Surgery, Peninsula Health, Frankston, VIC 3199, Australia; 2 Central Clinical School, Faculty of Medicine, Monash University, Melbourne, VIC 3004, Australia; 3 Plastic Surgery Unit, Department of Medicine, Surgery and Neuroscience, University of Siena, 53100 Siena, Italy

**Keywords:** AI, Generative Adversarial Networks, cosmetic surgery, rhinoplasty, facelifts, blepharoplasty

## Abstract

Artificial intelligence (AI), notably Generative Adversarial Networks, has the potential to transform medical and patient education. Leveraging GANs in medical fields, especially cosmetic surgery, provides a plethora of benefits, including upholding patient confidentiality, ensuring broad exposure to diverse patient scenarios, and democratizing medical education. This study investigated the capacity of AI models, DALL-E 2, Midjourney, and Blue Willow, to generate realistic images pertinent to cosmetic surgery. We combined the generative powers of ChatGPT-4 and Google’s BARD with these GANs to produce images of various noses, faces, and eyelids. Four board-certified plastic surgeons evaluated the generated images, eliminating the need for real patient photographs. Notably, generated images predominantly showcased female faces with lighter skin tones, lacking representation of males, older women, and those with a body mass index above 20. The integration of AI in cosmetic surgery offers enhanced patient education and training but demands careful and ethical incorporation to ensure comprehensive representation and uphold medical standards.

## 1. Introduction

Cosmetic surgery has undergone significant transformations over millennia, evolving with technological advancements and evolving societal standards. From the early procedures in ancient Egypt, India, and China, aimed primarily at reconstructing bodily damages and defects, the field has expanded to encompass both reconstructive and aesthetic dimensions, catering to a broad spectrum of needs and desires [1,2,3].

As modern medicine entered the digital age, the confluence of cosmetic surgery and Artificial Intelligence (AI) began to take shape. The rise of AI continues to reshape numerous sectors, notably medicine, research, and education [4]. Generative Adversarial Networks (GANs), an innovative subset of AI known for their prowess in image creation and analysis, have shown immense potential [5,6]. These systems harness vast databases and machine learning (ML) algorithms to discern statistical correlations between textual descriptions and corresponding images. Large language models (LLMs) use similar datasets to produce human-like responses based on user input and have shown promise in supporting clinical and academic medicine by aiding in diagnostic processes, augmenting traditional teaching, and research writing and data collection [7,8,9,10,11].

Such capabilities are transformative for generating hyper-realistic images pertinent to medical contexts. The integration of this technology is especially pivotal for fields like cosmetic surgery, where visualization and clinical representation play a central role [7,8,9,10,11]. Leveraging AI not only obviates the need for real patient images in educational environments, addressing both ethical and practical challenges, but also bolsters the safeguarding of patient confidentiality [9]. More significantly, medical education requires exposure to a myriad of patient profiles. AI-generated visuals ensure medical practitioners and students gain swift, comprehensive access to diverse patient scenarios. This diversity is paramount to molding adept healthcare professionals. Furthermore, the ubiquity of AI-generated images democratizes medical education [10]. Independent of geographic or financial barriers, learners worldwide can tap into identical, high-calibre educational content, thus leveling the educational playing field and fostering inclusive learning experiences. 

Cosmetic surgery, given its inherently visual nature, stands to gain profoundly from precise AI-generated imagery, offering students a rich reservoir of visual aids for comprehensive learning [11,12]. This amalgamation of technology and cosmetics can therefore foster a more in-depth understanding, potentially improving patient outcomes by better educating cosmetic surgeons. Our study specifically targets three core areas: the utility of AI-generated images as enriching educational tools for trainees; the capability of renowned AI models like DALL-E 2, Midjourney, and Blue Willow to produce clinically relevant, lifelike cosmetic surgery images; and the broader implications—including potential biases—of such AI advancements on cosmetic surgery education and practice. Through this lens, we aim to map out both the potential benefits and challenges of integrating AI into cosmetic surgery’s educational landscape.

## 2. Materials and Methods

**Figure 1:**
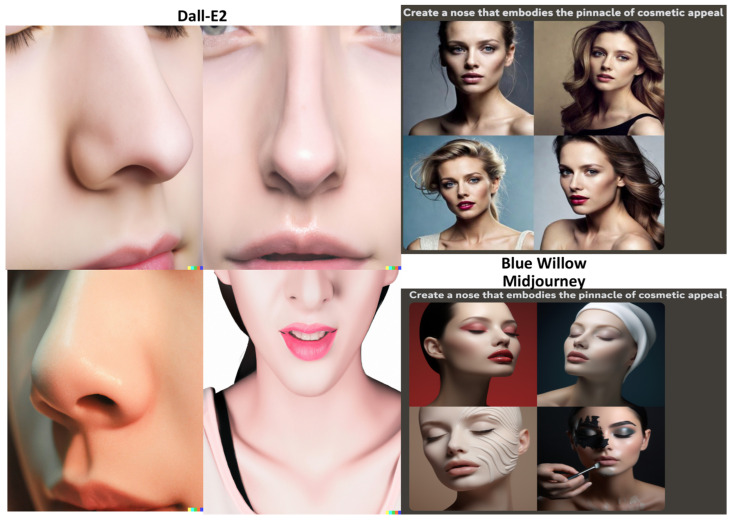
Results of the GANs for the prompt “Create a nose that embodies the pinnacle of cosmetic appeal”.

**Figure 2:**
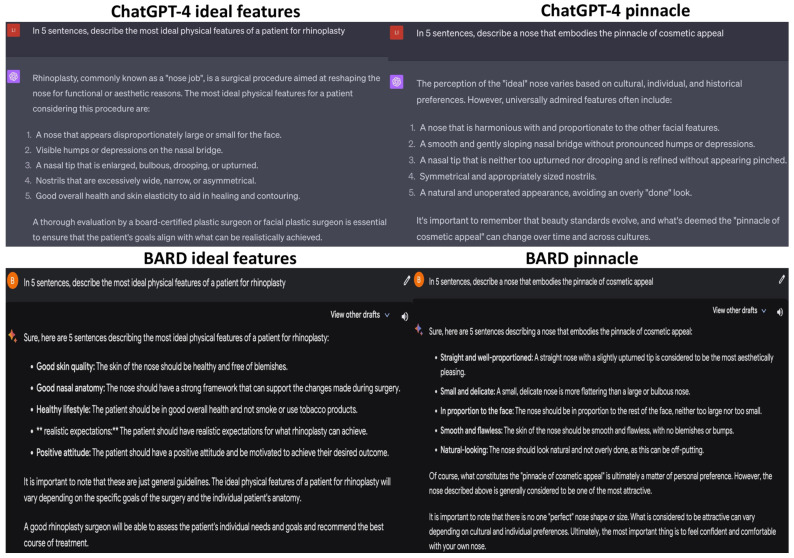
Results of the LLMs for the prompts “In five sentences, describe the most ideal physical features of a patient for rhinoplasty” and “In five sentences, describe a nose that embodies the pinnacle of cosmetic appeal”.

**Figure 3:**
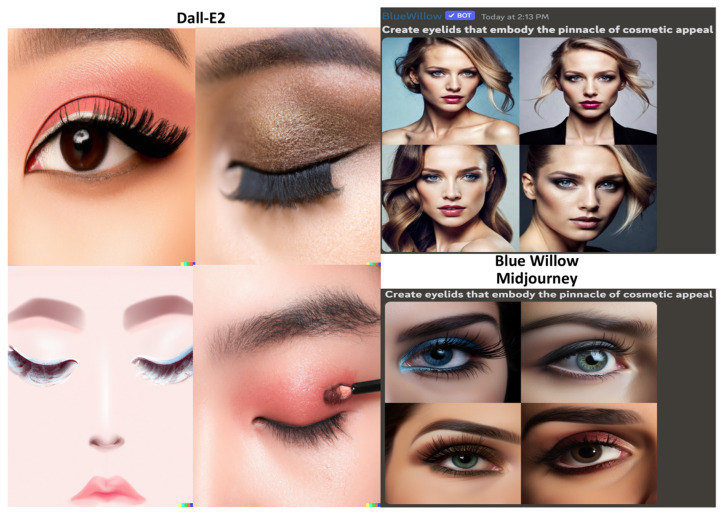
Results of the GANs for the prompt “Create eyelids that embody the pinnacle of cosmetic appeal”.

**Figure 4:**
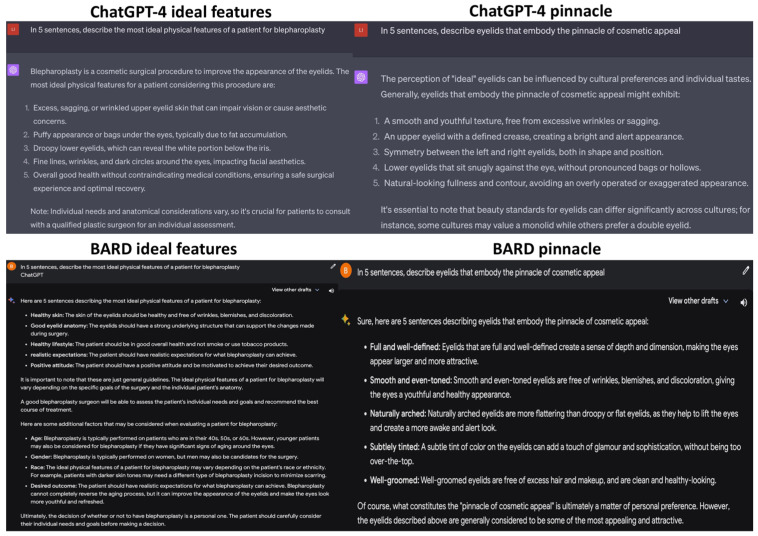
Results of the LLMs for the prompts “In five sentences, describe the most ideal physical features of a patient for blepharoplasty” and “In five sentences, describe eyelids that embody the pinnacle of cosmetic appeal”.

**Figure 5:**
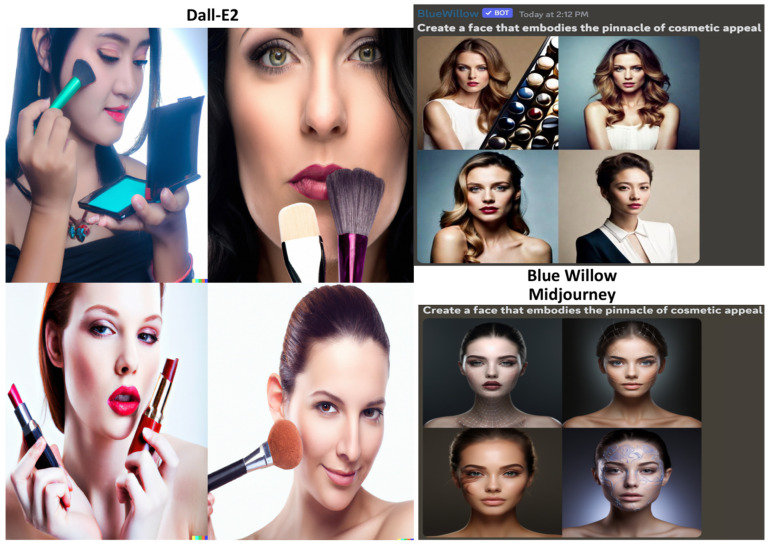
Results of the GANs for the prompt “Create a face that embodies the pinnacle of cosmetic appeal”.

**Figure 6:**
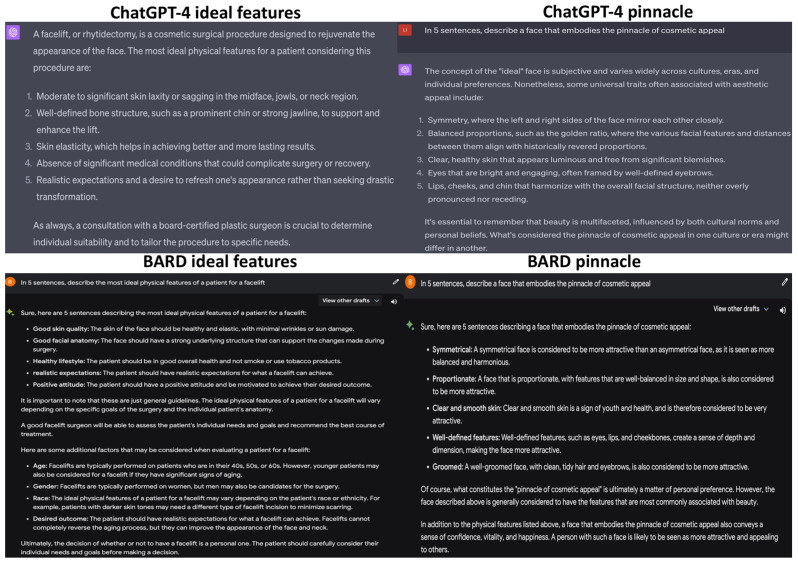
Results of the LLMs for the prompts “In five sentences, describe the most ideal physical features of a patient for a facelift” and “In five sentences, describe a face that embodies the pinnacle of cosmetic appeal”.

**Figure 7:**
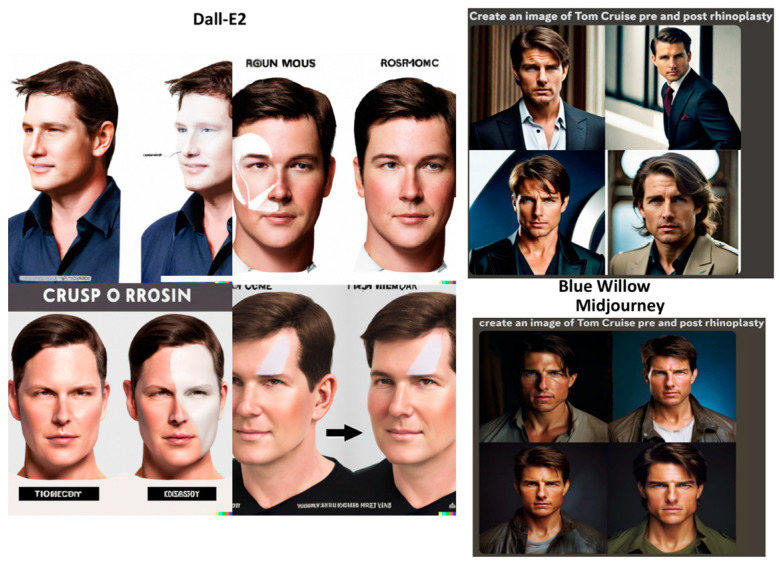
Results of the GANs for the prompt “Create an image of Tom Cruise pre and post rhinoplasty”.

**Figure 8:**
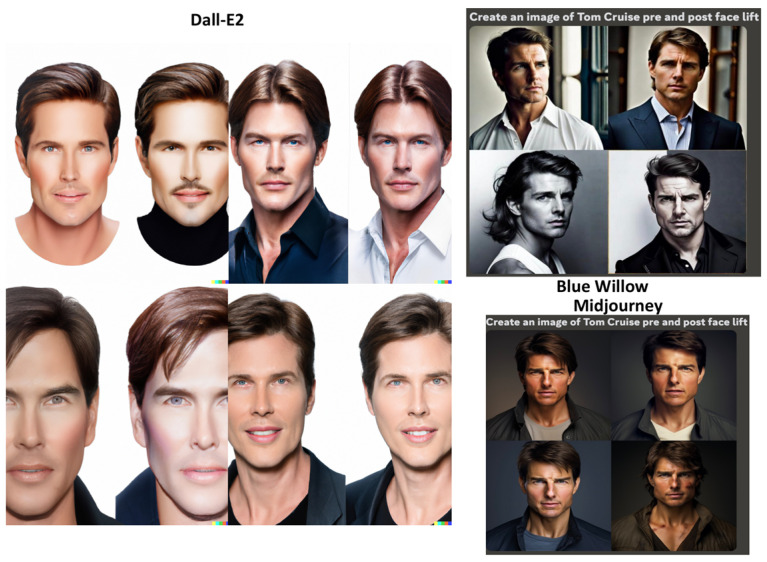
Results of the GANs for the prompt “Create an image of Tom Cruise pre and post face lift”.

**Figure 9:**
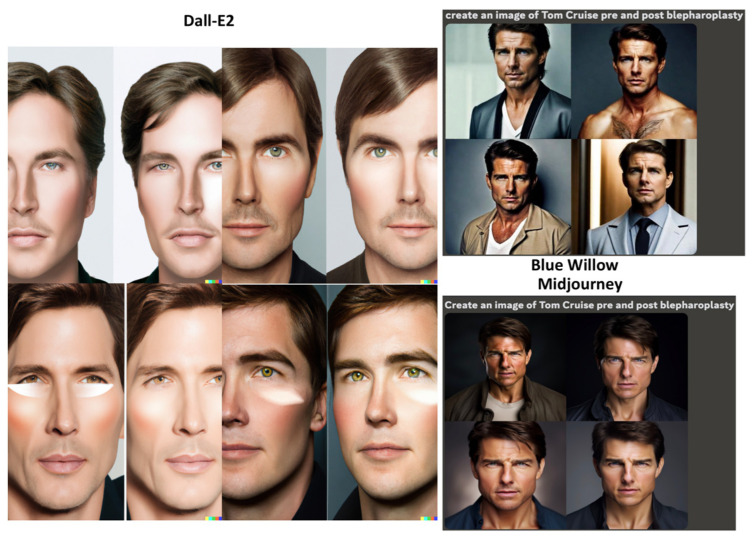
Results of the GANs for the prompt “Create an image of Tom Cruise pre and post blepharoplasty”.

In this study, we harnessed the generative capabilities of the LLMs ChatGPT-4 (https://chat.openai.com/, accessed 28 August 2023) and Google’s BARD (https://bard.google.com/, accessed 28 August 2023) in tandem with GANs DALL-E2 (https://openai.com/dall-e-2, accessed 28 August 2023), Midjourney (https://www.midjourney.com/, accessed 28 August 2023), and Blue Willow (https://www.bluewillow.ai/, accessed 28 August 2023) to craft images and descriptions representing the ideal standards of noses, faces, and eyelids. ChatGPT-4 and BARD utilize Transformer architectures to predict text sequences by being trained on vast bodies of information. The GANs consist of two adversarial networks—a generator that creates data and a discriminator that checks how realistic they look, enabling them to produce realistic synthetic images. Each LLM was provided with six distinct prompts, whereas each GAN received three. Subsequently, three experienced board-certified plastic surgeons (WMR, RJR, FS, and PC) appraised the outputs utilizing a Likert scale (Table 1), evaluating parameters such as comprehensibility, accuracy-to-real-life, and discernability. In cases of scoring disparities, discussions ensued until a unified agreement was established. Each prompt was constructed to exclude specific details such as age, gender, skin color, and make-up to assess the AI tools’ initial outputs, allowing for a more discerning evaluation of the AI tools’ inherent biases and limitations in their current programming. As our research solely employed publicly accessible AI-generated data, there was no mandate for institutional ethical clearance. The relevant figures (Figure 1, Figure 2, Figure 3, Figure 4, Figure 5, Figure 6, Figure 7, Figure 8 and Figure 9) are screenshots of the relevant outputs and their associated prompts.

## 3. Results

### 3.1. Rhinoplasty

The first prompt for the GANs read “Create a nose that embodies the pinnacle of cosmetic appeal”. Dall-E2, Midjourney, and Blue Willow (Figure 1) predominantly generated images of Caucasian women with conventionally attractive nasal features, such as ideal nasofrontal and nasolabial angles and nasal rotation without much dorsal hump or nasal deviations [13,14]. However, Dall-E2’s focus on the lower face hinders full nasal assessments, as rhinoplasty aims for overall facial harmony [15]. Unfortunately, all three GANs failed to produce images of the ala of the nose from the inferior aspect. The ala, especially the rim and base, hold significant relevance in terms of aesthetic standards [14].

ChatGPT and BARD were fed slightly different prompts: “In five sentences, describe the most ideal physical features of a patient for rhinoplasty” and “In five sentences, describe a nose that embodies the pinnacle of cosmetic appeal” (Figure 2). In response to the former prompt, ChatGPT accurately identified key aspects of the pre-operative assessment for a rhinoplasty. However, it did not structure its analysis by acquiring frontal, lateral, and basal views for inspection or include palpation assessments for thickness, pliability, and texture [16]. ChatGPT’s answer to the second prompt used simple terms and addressed valid factors, but unfortunately omitted technical details like nasal tip angulation and geometrical polygons essential for a plastic surgeon [17]. BARD’s answer to the first prompt was lower in quality, mentioning two valid factors out of five. This suggests that it misunderstood the prompt, as the other three factors referenced patient attitudes rather than their physical appearance. BARD’s response to the second prompt mirrored ChatGPT’s, with both concluding that nasal beauty standards change across time and cultures.

### 3.2. Blepharoplasty

The second prompt for the GANs read, “Create eyelids that embody the pinnacle of cosmetic appeal” (Figure 3). All three GANs again produced similarly-appearing double eyelids that boast darker shades, long eyelashes, and middle-height crease levels [18]. Dall-E2 and Midjourney showcased cropped eyelid images, whereas Blue Willow produced full-face photos, making it superior for evaluating eyelid aesthetics. However, Blue Willow’s images were limited to Caucasian women, narrowing the scope of our analysis to one skin tone and gender. 

The LLMs were prompted, “In five sentences, describe the most ideal physical features of a patient for blepharoplasty” and “In five sentences, describe eyelids that embody the pinnacle of cosmetic appeal” (Figure 4). ChatGPT accurately listed common physical features that are ideal for blepharoplasty [19]. However, its mention of a patient’s overall health deviated from the prompt, focusing instead on physiological health. It also accurately highlighted key features that people tend to look for in beautiful eyelids [18,19,20]. Analogous to its previous response, BARD listed two out of five ideal physical features for blepharoplasty and expanded on supplementary characteristics like age, gender, and desired outcomes, which ChatGPT missed. BARD delineated similar points as ChatGPT did when answering the latter prompt. Both LLMs conclude their responses by reiterating that beauty standards are subjective, but highlighted features are often deemed most attractive.

### 3.3. Facelift

The third prompt to the GANs read “Create a face that embodies the pinnacle of cosmetic appeal”. Dall-E2 created the most realistic images of women’s faces, often featuring makeup applications, followed by Blue Willow and then Midjourney, which added a sci-fi element and offered only frontal views (Figure 5). All three GANs depicted symmetrically round faces with lighter skin tones, voluminous lips, smooth complexions, and lower facial adiposity levels [21,22,23].

The LLMs were fed a third set of prompts: “In five sentences, describe the most ideal physical features of a patient for a facelift” and “In five sentences, describe a face that embodies the pinnacle of cosmetic appeal” (Figure 6). ChatGPT’s response, similar to its previous answers, listed five ideal features for facelifts. However, only three described physical traits, while the last two addressed patient attitudes and expectations. In response to the second prompt, it accurately delineated several traits commonly associated with aesthetic appeal in the realm of cosmetic considerations [21,24]. BARD, similar to before, listed five ideal features, but only two pertained to pure physical appearance, while the rest focused on patient attitudes towards facelift surgery. The facial features it described as conventionally attractive paralleled those mentioned by ChatGPT.

### 3.4. Potential Bias

It is noteworthy that the imagery produced by all three GANs exclusively represented female faces, with an absence of male representation. The depicted females predominantly, if not exclusively, exhibited lighter skin tones. Additionally, there was an apparent absence of representations of women presumed to be above 50 years of age or with a Body Mass Index (BMI) exceeding 20 (Table 2).

### 3.5. Celebrity Faces

The GANs were prompted to: “Create an image of Tom Cruise pre and post rhinoplasty”, “Create an image of Tom Cruise pre and post face lift”, and “Create an image of Tom Cruise pre and post blepharoplasty” (Figure 7, Figure 8 and Figure 9). In the Dall-E2-generated imagery, post-operative representations predominantly showcased widened ala and more defined nasal tips for rhinoplasty procedures. Surprisingly, post-operative facelift visuals depicted lighter skin tones. Blepharoplasty images exhibited minimally significant alterations. Notably, Dall-E2’s depictions labeled “Tom Cruise” were inconsistent, presenting a face not recognizable as the actor but rather a random male’s. Another limitation is that many images did not display the full face, impeding evaluation of the operation’s effects. Blue Willow’s imagery exhibited superior quality, presenting facial resemblances more akin to Tom Cruise. While the platform provided comprehensive facial visuals, facilitating post-operative analysis, the excessive zoom diminished the capacity for detailed examination. Additionally, the suboptimal lighting conditions compromised the discernibility of finer details. Midjourney produced the most authentic facial representations, closely mirroring Tom Cruise. However, subdued lighting conditions slightly obscured facial details. The differentiation between pre- and post-operative images remained minimal.

## 4. Discussion

Cosmetic surgery stands out as a field intricately tied to visual representation. The prospect of integrating AI-generated images in this domain can profoundly reshape its paradigms, especially concerning patient counseling, education, and training.

In evaluating GAN outputs for rhinoplasty, blepharoplasty, and facelift prompts, prevalent biases were evident: Dall-E2, Midjourney, and Blue Willow predominantly highlighted Caucasian women with traditional beauty standards. Dall-E2’s images for rhinoplasty lacked comprehensive views; they specifically showcased just the eyelids, whereas Blue Willow’s full-face images offered a broader assessment but were limited demographically. Facelift outputs, particularly from Dall-E2, were realistic and consistent with conventional beauty standards, but the collective leaning towards lighter skin tones and certain facial features suggests potential limitations in training data and raises questions about ingrained societal beauty norms.

In terms of potential educational use in cosmetic analysis, the GANs suffer jarring limitations. Midjourney’s pseudo-realistic, artsy renderings diverge from lifelike representations, hindering its utility. Dall-E2’s emphasis on specific organs, although detailed, precludes a holistic appreciation of the face, which is essential for cosmetic evaluations. Although Blue Willow demonstrated the most promising results, its lack of multiple angles (lateral, inferior, and oblique) restricts comprehensive assessment. Further, the collective bias towards lighter skin tones skews the analysis, suggesting these GANs currently fall short of offering an equitable and rounded educational tool in the realm of cosmetic surgery.

The amalgamation of AI with cosmetic surgery offers myriad benefits for patient engagement and understanding. AI’s unparalleled ability to generate tailored images customized to patient descriptions paves the way for a heightened personalized visualization experience. Imagine a patient’s ability to see, beforehand, an AI-projected image of their postoperative outcome. Such visual aids are not merely illustrative; they play a vital role in setting and managing expectations, offering patients tangible insight into potential outcomes [11,25,26]. By facilitating clearer visual comprehension, AI empowers patients to make more informed decisions regarding their procedures [25,27]. Additionally, the technology can cater to individual concerns, illustrating everything from the nuances of facial symmetry post-rhinoplasty to the trajectory of scar healing [26]. The inherent advantage here is twofold: not only do patients gain a clearer perspective, but this clarity also alleviates potential anxieties. From an ethical standpoint, the use of AI-generated images mitigates the complexities surrounding the use of real patient images, striking a balance between education and privacy [28,29]. The advanced accuracy of AI tools in predicting post-operative images, such as Vectra and Crisalix, can paradoxically create unrealistic patient expectations, leading to patient dissatisfaction if outcomes differ. Surgical results can be affected by anatomical and technical variables, with surgeons sometimes adjusting plans mid-operation. Therefore, it might be more practical for AI to suggest potential outcomes, followed by a discussion of what is realistically achievable.

For budding cosmetic surgeons, AI-generated images present a transformative learning tool. Medical education, particularly in specialized fields like cosmetic surgery, demands exposure to a plethora of cases. Here, AI shines by offering visual representations of even the rarest of cases, ensuring trainees receive a well-rounded, comprehensive education. Unlike static traditional images, AI’s dynamic visual generation—tailored to specific queries or areas of academic intrigue—promotes an interactive, engaged learning environment. This dynamism not only enriches the learning experience but also addresses ethical challenges, notably those related to patient consent and confidentiality. A unique benefit is the standardization AI brings. With it, there is an assurance that all trainees, irrespective of geographical or institutional divides, will access uniformly high-caliber visual resources. This uniformity is pivotal in championing a global gold standard in cosmetic surgery education. As the medical field evolves, AI’s adaptability ensures that trainees are always in sync with the latest procedures and techniques.

However, the marriage of AI and cosmetic surgery is not without challenges. Based on their training data, there is an inherent risk of them mirroring and perpetuating biases, potentially leading to skewed or non-representative visual outputs [30,31,32,33]. A critical balance is paramount; while AI is undeniably a potent tool, it must not overshadow or replace the intrinsic value of hands-on experience and direct patient interaction. Further, continuous validation is essential to guaranteeing the medical and scientific accuracy of AI-produced images. Moreover, the GANs produce images favoring younger aesthetics, despite older individuals typically seeking procedures like blepharoplasties and facelifts. This suggests GANs may not fully grasp the societal context and primary audience for such procedures. For GANs to be invaluable in cosmetic surgery, they must be trained on diverse datasets covering various ethnicities, ages, and demographics. Such inclusive training ensures AI-generated images accurately reflect the broad spectrum of those seeking cosmetic procedures, benefiting both medical education and patient consultations. It is vital that these technologies honor diverse beauty standards and advance in tandem with an ethos of inclusivity, ensuring no demographic remains overlooked.

The challenges posed by the integration of AI in cosmetic surgery, particularly those concerning potential biases and the risk of over-reliance, necessitate proactive solutions. One primary solution could be the establishment of protocols for the regular validation of AI tools. This would entail frequent benchmarking against real-world outcomes to gauge accuracy and recalibrate algorithms as needed. Additionally, the curriculum for medical training should incorporate a balance between AI-enhanced learning and hands-on clinical experience. While AI can provide a vast reservoir of visual aids and theoretical knowledge, hands-on practice under expert supervision remains irreplaceable. Emphasizing the complementary nature of AI tools rather than viewing them as replacements can mitigate the risk of over-reliance.

Regarding regulations, the rapid advancement of AI in cosmetic surgery calls for robust oversight by both governmental bodies and industry stakeholders. As AI technologies become increasingly ingrained in the field of cosmetic surgery, the urgency of implementing standardized ethical guidelines has never been more acute. These guidelines must focus on multiple dimensions of ethical concerns: safeguarding patient confidentiality, ensuring transparency in AI-driven decision-making processes, and offering avenues for redress in case of errors attributable to AI. Beyond these, it is crucial for ethical frameworks to also tackle issues related to representation and inclusivity. Specifically, AI tools should be trained on diverse datasets to mitigate the risk of perpetuating existing biases. To develop such a multi-faceted framework, it is imperative to foster collaborative efforts among various stakeholders. This includes governmental regulatory bodies, industry pioneers, and medical professionals. Through collective action, we can create an expansive set of guidelines that not only encourages innovation but also rigorously upholds ethical standards, patient welfare, and the broader integrity of the cosmetic surgery field.

## 5. Conclusions

In summary, the horizons of cosmetic surgery are broadening with the advent of AI-generated imagery. This merger promises enriched patient education, more effective counseling, and an enhanced training paradigm. Nevertheless, as with any technological innovation, its adoption should be judicious, ensuring it augments existing methodologies rather than replacing them. The juxtaposition of innovation and ethics in this realm also necessitates rigorous oversight and regular updates.

## Figures and Tables

**Table 1:** Likert scale for evaluation of GANs.

Criteria	DALL-E	Midjourney	Blue Willow
The AI-generated images resemble traditional real-world beauty standards	[ ] 1—Strongly Disagree [ ] 2—Disagree [ ] 3—Neither Agree or Disagree [x] 4—Agree [ ] 5—Strongly Agree	[ ] 1—Strongly Disagree [ ] 2—Disagree [x] 3—Neither Agree or Disagree [ ] 4—Agree [ ] 5—Strongly Agree	[ ] 1—Strongly Disagree [ ] 2—Disagree [ ] 3—Neither Agree or Disagree [x] 4—Agree [ ] 5—Strongly Agree
The AI-generated images adequately represent the specific organ(s)	[ ] 1—Strongly Disagree [ ] 2—Disagree [ ] 3—Neither Agree or Disagree [x] 4—Agree [ ] 5—Strongly Agree	[ ] 1—Strongly Disagree [ ] 2—Disagree [ ] 3—Neither Agree or Disagree [x] 4—Agree [ ] 5—Strongly Agree	[ ] 1—Strongly Disagree [ ] 2—Disagree [ ] 3—Neither Agree or Disagree [x] 4—Agree [ ] 5—Strongly Agree
The AI-generated images’ details are visible and easily discernible	[ ] 1—Strongly Disagree [ ] 2—Disagree [x] 3—Neither Agree or Disagree [ ] 4—Agree [ ] 5—Strongly Agree	[ ] 1—Strongly Disagree [ ] 2—Disagree [x] 3—Neither Agree or Disagree [ ] 4—Agree [ ] 5—Strongly Agree	[x] 1—Strongly Disagree [ ] 2—Disagree [ ] 3—Neither Agree or Disagree [x] 4—Agree [ ] 5—Strongly Agree
The AI-generated images are of high quality	[ ] 1—Strongly Disagree [ ] 2—Disagree [x] 3—Neither Agree or Disagree [ ] 4—Agree [ ] 5—Strongly Agree	[ ] 1—Strongly Disagree [ ] 2—Disagree [x] 3—Neither Agree or Disagree [ ] 4—Agree [ ] 5—Strongly Agree	[ ] 1—Strongly Disagree [ ] 2—Disagree [ ] 3—Neither Agree or Disagree [x] 4—Agree [ ] 5—Strongly Agree
The AI-generated images are beneficial for educational purposes	[ ] 1—Strongly Disagree [x] 2—Disagree [ ] 3—Neither Agree or Disagree [ ] 4—Agree [ ] 5—Strongly Agree	[ ] 1—Strongly Disagree [x] 2—Disagree [ ] 3—Neither Agree or Disagree [ ] 4—Agree [ ] 5—Strongly Agree	[ ] 1—Strongly Disagree [x] 2—Disagree [ ] 3—Neither Agree or Disagree [ ] 4—Agree [ ] 5—Strongly Agree

**Table 2:** Percentages of various characteristics of noses, faces, and eyelids depicted by GANs.

	Characteristic		Gender Male Female	Skin Tone White/Caucasian Other	Age (Assumed) <50 yrs >50 yrs	BMI (Assumed) <20 >20
Nose	Number (%)	Dall-E2	0 (0%) 4 (100%)	3 (75%) 1 (25%)	4 (100%) 0 (0%)	4 (100%) 0 (0%)
		Midjourney	0 (0%) 4 (100%)	4 (100%) 0 (0%)	4 (100%) 0 (0%)	4 (100%) 0 (0%)
		Blue Willow	0 (0%) 4 (100%)	4 (100%) 0 (0%)	4 (100%) 0 (0%)	4 (100%) 0 (0%)
Face	Characteristic					
	Number (%)	Dall-E2	0 (0%) 4 (100%)	3 (75%) 1 (25%)	4 (100%) 0 (0%)	4 (100%) 0 (0%)
		Midjourney	0 (0%) 4 (100%)	4 (100%) 0 (0%)	4 (100%) 0 (0%)	4 (100%) 0 (0%)
		Blue Willow	0 (0%) 4 (100%)	3 (75%) 1 (25%)	4 (100%) 0 (0%)	4 (100%) 0 (0%)
Eyelids	Characteristic					
	Number (%)	Dall-E2	0 (0%) 4 (100%)	Unable to determine	4 (100%) 0 (0%)	4 (100%) 0 (0%)
		Midjourney	0 (0%) 4 (100%)	3 (75%) 1 (25%)	4 (100%) 0 (0%)	4 (100%) 0 (0%)
		Blue Willow	0 (0%) 4 (100%)	4 (100%) 0 (0%)	4 (100%) 0 (0%)	4 (100%) 0 (0%)

## Data Availability

The authors confirm that the data supporting the findings of this study are available within the article.
